# Gender differences in the association between cigarette smoking, alcohol consumption and depressive symptoms: a cross-sectional study among Chinese adolescents

**DOI:** 10.1038/srep17959

**Published:** 2015-12-07

**Authors:** Yue Yue, Lingyao Hong, Lan Guo, Xue Gao, Jianxiong Deng, Jinghui Huang, Guoliang Huang, Ciyong Lu

**Affiliations:** 1 School of Public Health, Sun Yat-sen University, Department of Medical Statistics and Epidemiology, Guangzhou, 510000, China; 2 The First Affiliated Hospital, Sun Yat-sen University, Epidemiology Research Unit, Clinical Trials Center, Guangzhou, 510000, China; 3 Centre for ADR Monitoring of Guangdong, Guangzhou, 510000, China

## Abstract

The aim of this study was to examine the association between cigarette smoking, alcohol consumption and depressive symptoms among adolescents, with a particular focus on gender differences. A total of 19,578 middle and high school students in Chongqing Province were surveyed. Self-reported cigarette smoking, alcohol consumption, depressive symptoms, and family- and school-related factors were assessed. A total of 8.8% adolescents reported smoking cigarettes. Tobacco use by boys (16.5%) was significantly higher than by girls (1.9%). Approximately 23.5% of adolescents reported alcohol consumption. Consumption in boys (31.5%) was significantly higher than in girls (16.2%). Depressive symptoms were prevalent in 9.1% of the sample. Girls reported significantly more symptoms (10.4%) than boys (7.7%). Multiple logistic regression analyses showed that the association between alcohol consumption and depressive symptoms was stronger among girls (AOR = 2.1, 95% CI = 1.8–2.5) than boys (AOR = 1.7, 95% CI = 1.4–2.1). A significant association (AOR = 2.3, 95% CI = 1.6–3.4) between cigarette smoking and depressive symptoms was revealed in girls only. The significant gender differences found above may provide a basis for the early identification of individuals at high risk for depression.

The transitional stage between childhood and adulthood known as adolescence, is a critical time for physical and mental development. Many health risk behaviors, such as depression, smoking, and drinking, tend to occur in this phase. Depression is the predominant cause of illness and disability for both boys and girls aged 10 to 19, according to the World Health Organization’s (WHO’s) 2014 “Health for the world’s adolescents” report^1^. Meta-analyses suggest the prevalence of depressive disorder in adolescents is 5.7%^2^. Adolescence and young adulthood are associated with high rates of new depression cases^3^. Depression negatively impacts growth, development, school performance, and peer and family relationships. Adolescents diagnosed with depressive disorders are at higher risk of substance abuse, future depression, and suicidal behavior^4^. Additionally, research has shown that most long-term adult smokers start smoking during adolescence^5^. According to national surveillance in the United States, 15.7% of high school students had smoked cigarettes during the 30 days prior to the survey^6^. Approximately one in ten Canadian high school students are current smokers^7^. Further, 34.9% of American high school students had consumed alcohol during the 30 days prior to the survey^6^. Excessive alcohol consumption in adolescents is strongly related to problem behavior and increased risk of suicidal behavior^8^,^9^.

Recent studies focused on the directions of the associations among smoking, drinking and depression have been unclear. Depression can potentially cause smoking or drinking. This relationship can be explained by the self-medication theory, which states that drinking and smoking can elevate mood and relieve depressed feelings^10^,^11^. Two longitudinal studies showed that depression may lead to the initiation of alcohol consumption for boys^12^,^13^. Smoking or drinking can also lead to depression. Alcohol toxic effect on the thyroid gland can potentially lead to hypothyroidism, which is associated with clinical depression^14^. Nicotine affects neurotransmitter activity in the brain and can cause changes that increase the risk of depression^15^. The relationship may even be bidirectional. A prospective cohort study found a bidirectional relationship between adolescent depression and smoking^16^. The relationship may be caused by shared risk factors (possibly genetic), in which no causality exist^17^. Roy *et al.*^18^ found no difference between smokers and non-smokers in history or severity of depression. They concluded that cigarette smoking and depression were related based on early deprivation variables, rather than one causing the other. Rohde and colleagues^19^ failed to find such association. They found that externalizing disorders, except for depression, were associated with daily smoking.

Some evidence has suggested that the association between tobacco, alcohol and depression may differ by genders. However, findings are inconsistent, and limited research has conducted among adolescents. Males have demonstrated stronger associations than females. Depression levels were associated with smoking statuses of 14- to 18-year-old adolescent boys, but not girls^20^. Men who drink heavily at least four times during the previous 28 days had a 2.6-fold higher risk for depression, which was not found in women^21^. Other studies have demonstrated opposite findings. Adolescent 12- to 17-year-old girls in the U.S with a diagnosis of depression were two times more likely to smoke, but boys did not show a similar association^22^. Depressive disorder was positively associated with later alcohol use disorders (OR = 3.11) in women, but not in men, in a longitudinal study^23^. Having more than 5 drinks on a maximal drinking occasion may be associated with increased risk of major depression among women, according to a follow-up study^24^. Prior diagnosis of major depression significantly affected risk for developing alcohol dependence in women, according to a longitudinal study^25^.

Smoking and drinking are common phenomena among adolescents, and depression can cause a series of negative consequences. Most previous studies regarding the association between health risk behaviors and gender differences have been conducted in western or developed countries. Very few have been conducted in developing countries, such as China. Furthermore, research has not been consistent regarding gender differences in this association. Therefore, we conducted a large-scale cross-sectional study among Chinese adolescents to examine the association between cigarette smoking, alcohol consumption and depressive symptoms, with a particular focus on gender differences.

The following two hypotheses were formulated. First, we hypothesized that cigarette smoking and alcohol consumption would be significantly correlated with depressive symptoms. Second, we hypothesized that the relationship between cigarette smoking, alcohol consumption and depressive symptoms would significantly differ by gender.

## Results

A total of 19,578 students were invited to participate, of which 18496 (94.5%) student questionnaires were completed and included for analysis. Those who were excluded were similar to the sample we analyzed in terms of demographics (data not shown). The participants’ characteristics are summarized in Table 1. Boys accounted for 47.4% (8765) of the sample. Students were from 12 to 19 years old, with a mean age of 15.5 (±1.7) years. Approximately 54.7% (10123) of the students lived with both biological parents, whereas 16.4% (3036) lived in single-parent families. Approximately 10.5% (1944) of students reported their family economic status to be above average, whereas 31.7% (5857) reported below average. Approximately 42.0% (7760) of the students communicated little with parents. A total of 3.4% (635) of participants reported poor relationships with teachers, while 7.2% (1325) had poor relations with classmates. Students who thought their academic pressure was above average accounted for 42.5% (7859) of the sample. Correlations between these categorical variables and cigarette smoking, alcohol consumption and depressive symptoms were analyzed by Chi-square tests.

According to the CES-D, approximately 9.1% (1685) of adolescents in Chongqing had depressive symptoms. The mean score for the entire sample was 15.2 (±9.0). Additionally, 10.4% (1012) of girls had depressive symptoms, with a mean score of 15.9 (±9.2). Approximately 7.7% (673) of boys had depressive symptoms, with a mean score of 14.4 (±8.7). A significant difference existed between boys and girls (χ^2^ = 41.2, *P* < 0.01).

During the 30 days prior to the survey, approximately 8.8% (1636) of the adolescents reported smoking cigarettes. Use was significantly higher in boys (16.5%) than girls (1.9%) (χ^2^ = 1224.4, *P* < 0.01). Among adolescent smokers, 49.0% smoked their first entire cigarette before they were 13 years old. No significant difference between boys (51.4%) and girls (46.4%) existed for this proportion (χ^2^ = 1.6, *P* = 0.2).

During the 30 days prior to the survey, approximately 23.5% (4340) of adolescents reported alcohol consumption. Use was significantly higher in boys (31.5%) than girls (16.2%) (χ^2^ = 597.3, *P* < 0.01). Among adolescent drinkers, 63.9% drank for the first time before age 13. Such drinking significantly higher in boys (72.5%) than girls (59.7%) (χ^2^ = 203.3, *P* < 0.01).

Table 1 shows the values for the CIE (change in estimate) of each covariate being added to the crude model. CIE^1^ means the change in estimate of the association between cigarette smoking and depressive symptoms resulting from including the covariate into the crude model. A simulated cutoff of 0.3% was found to achieve a significance level of 5% in the association between cigarette smoking and depressive symptoms using R V.3.2.2. In examining the list of potential confounders, the change in the estimate was larger than 0.3% for all variables except living arrangement (−0.2%). CIE^2^ means the change in estimate of the association between alcohol consumption and depressive symptoms resulting from including the covariate into the crude model. A simulated cutoff of 0.06% was found to achieve a significance level of 5% in the association between alcohol consumption and depressive symptoms. In examining the list of potential confounders, the change in the estimate was larger than 0.06% for all variables except living arrangement (0%). Thus the following covariates were considered to be confounders adjusted for the multiple logistic regression: grade, family economic status, parental education level, parental smoking and drinking pattern, communication with parents, number of brothers and sisters, number of good friends, relationship with teachers and classmates, pocket money, academic pressure and time doing homework.

The final logistic regression model for having depressive symptoms is presented in Table 2. After controlling for confounders, the interaction between cigarette smoking and gender was significant (P < 0.001). The interaction between alcohol consumption and gender was also significant (P = 0.007). The stratified analysis by sex showed that cigarette smoking did not have a significant association with depressive symptoms for boys, after controlling for confounders. Alcohol consumption was shown to be a risk factor for having depressive symptoms among boys (AOR = 1.7, 95% CI = 1.4-2.1). For girls, both cigarette smoking (AOR = 2.3, 95% CI = 1.6-3.4) and alcohol consumption (AOR = 2.1, 95% CI = 1.8-2.5) were risk factors for having depressive symptoms after controlling for confounders.

## Discussion

We found that 8.8% of adolescents in Chongqing reported smoking cigarettes. Approximately 23.5% reported alcohol consumption and 9.1% had depressive symptoms. The proportion of cigarette smoking in boys (16.5%) was significantly higher than in girls (1.9%). The proportion of boys who drank alcohol (31.5%) was also significantly higher than girls (16.2%). However, the proportion of girls with depressive symptoms (10.4%) was significantly higher than boys (7.7%). These findings confirm previous studies that males are more likely to have abused, or been dependent on, substances^26^,^27^. Females are more likely to have depressive symptoms^28^. Tobacco and alcohol use in boys reflects their newly gained independence. However, girls who smoke or drink are not always socially accepted in China. Girls are also more emotional than boys and may be more vulnerable to mental illness.

After controlling for confounding factors, the association between alcohol consumption and depressive symptoms was stronger among girls (AOR = 2.1, 95% CI = 1.8–2.5) than boys (AOR = 1.7, 95% CI = 1.4–2.1). This finding is consistent with another cross-sectional study showing that binge drinking and mental health problems were more common among 12- to 15-year-old adolescent girls (OR = 2.43) than boys (OR = 1.64)^29^. The 2010 National Survey on Drug Use and Health in American college students showed that the association between alcohol abuse and mental illness was stronger among females than males^30^.

This study found that cigarette smoking and depressive symptoms were significantly associated in girls only (AOR = 2.3, 95% CI = 1.6–3.4). This result slightly disagrees with a longitudinal follow-up survey performed in adolescents aged 12 to 18 in the U.S.^28^, showing that smoking status was a significant predictor of developing notable depressive symptoms in both genders. However, the effect in girls (OR = 2.05, 95% CI = 1.39–3.04) was greater than boys (OR = 1.86, 95% CI = 1.18–2.92). Although these two studies showed a stronger association in girls, the variation in boys should be considered. This variation may be driven by many reasons including different time periods, target populations, study design and methodological definitions of cigarette smoking, alcohol consumption and depressive symptoms. Further large meta-analysis or pool analyses are warranted to explore the potential correlates of heterogeneity of findings existing in current literature.

Cigarette smoking and alcohol consumption were shown to be associated with depressive symptoms in girls, which is consistent with our first hypothesis. Moreover, our study showed gender differences in this association, which is consistent with our second hypothesis.

Recent evidence suggests a biological plausibility of the gender differential association between cigarette smoking, alcohol consumption and depressive symptoms. Women may experience more detrimental effects from alcohol consumption due to metabolic factors such as decreased alcohol dehydrogenase, an enzyme used to metabolize alcohol^31^. Women of all ages have less lean muscle mass than men, making them more susceptible to the negative effects of alcohol^32^. Additionally, women may be more sensitive to harmful effects of smoking because of interactions between components of smoke and hormones^33^. Generally speaking, smoking and drinking pose greater health risks for women in terms of various physical and mental factors than for their male counterparts. Therefore, smoking and drinking associated more with depressive symptoms in females.

Our study has several strengths. First, to our knowledge no research has specifically and comprehensively considered gender difference in the association between cigarette smoking, alcohol consumption and depressive symptoms in terms of demographics and school and family domains in this population. We conducted this survey in China and found a gender differential association. Second, our study used a large and randomly selected sample of Chinese adolescents, which renders us sufficient statistical power to detect possible associations even after adjusting for a considerable of potential confounders. However, several limitations were present. First, the cross-sectional design limited the ability to confirm the temporal sequence. Therefore, we failed to draw conclusions about causality. Longitudinal investigations are necessary in subsequent studies. Second, the CES-D is not a tool for diagnosing clinical depression but a screening instrument to assess the frequency of depressive symptoms. Cigarette smoking may be associated with clinical depression in boys. Third, results were based on a structured self-rating questionnaire. Although self-reporting is a common and accepted research method, students’ responses to sensitive questions about undesirable behavior may be biased. We attempted to assure that the study was performed anonymously to encourage participants to respond truthfully.

In conclusion, our study shows that the association between cigarette smoking, alcohol consumption and depressive symptoms is stronger in girls than boys in a developing country. This finding is an important addition to existing literature. By examining this association, this study may support the early identification of individuals at high risk for depression. We may pay more attention to drinking and smoking adolescents, especially girls, to detect depressive symptoms and offer the help in time. This early identification may be of practical importance for both adolescents and guardians. Our study shows gender differences in the association between cigarette smoking, alcohol consumption and depressive symptoms. However, no causal relationship can be established from this cross-sectional design. We hope future longitudinal studies account for our findings and that policy makers utilize this information to take gender specific measures.

## Methods

### Study design and participants

This cross-sectional study was based on citywide representative sample. Participants were middle and high school students from Chongqing, an inland super-large city of China. A multistage stratified-cluster random-sampling method was used to select participants. In stage 1, we divided the entire city into three levels according to economic status (most developed, medium developed and least developed). Then, we randomly selected two counties from each level. In stage 2, schools were divided into three categories: middle (grades 7–9), high (grades 10–12) and vocational high schools (grades 10–12). We randomly selected three types of schools from each county. Six middle, four high and two vocational high schools were selected from each county. In stage 3, two classes were randomly selected from each grade in these schools. All available students within the class were surveyed. Those not surveyed were absent or refused to participate and consisted of less than 1% of the student population. The self-report questionnaire was designed by our expert group. Criterion and instrumentation proposed by the WHO were adopted. We carried out a pilot investigation to adapt questions to Chinese. Many previous studies have taken similar translation steps^34^,^35^. Data collection was anonymous. Questionnaires were administered in classrooms, by research assistants and without the presence of the teachers. All data were collected in 2012.

### Ethical statement

The study received approval from the Sun Yat-Sen University School of Public Health Institutional Review Board. All participants were fully informed of the purpose of the study and invited to participate voluntarily. Written consent letters were obtained from the school, each participating student and one of each student’s parents. Methods were carried out in accordance with the approved guidelines.

### Measures

#### Cigarette smoking

Cigarette smoking was defined as more than one cigarette at least one day in the preceding 30 days. The age of initial smoking was measured by asking “How old were you when you first smoked an entire cigarette?” Similar definitions of cigarette smoking have been used in previous studies^36^,^37^.

#### Alcohol consumption

Alcohol consumption was defined as more than one drink (a can or bottle of beer, a glass of wine or a wine cooler, a shot of liquor, or a mixed drink with liquor in it) at least one day in the preceding 30 days. The age of initial alcohol consumption was measured by asking “How old were you when you first consumed alcohol”? Similar definitions of alcohol consumption have been used in previous studies^38^,^39^.

#### Depressive symptoms

The Center for Epidemiology Scale for Depression (CES-D) in Chinese was used to identify depressive symptoms. Respondents were asked to rate the frequency of 20 depressive symptoms within the past week on a scale of 1 of to 4, ranging from ‘rarely or none of the time’ to ‘most or all of the time’^40^. The total score ranges from 0 to 60. Higher scores indicate higher level of depressive symptoms. The original cut-off score for depressive symptoms was 16 points (corresponding to the 80th percentiles) in 1977^41^. A score of 28 points (corresponding to the 95th percentiles) was later used to select more severe cases^42^. We adopted 28 points as the cut-off in our survey. Those who failed to answer at least 17 of the 20 items were eliminated. The CES-D has been shown to have high internal consistency, test–retest reliability, and concurrent and construct validity. The instrument has been used in adolescents^42^,^43^,^44^. The Chinese version of this scale has also been validated^45^,^46^,^47^ and extensively utilized in Chinese studies^48^.

#### Sociodemographics and family- and school-related factors

Relevant variables include gender, age, grade, living arrangement, family economic status, parental education level, parental smoking and drinking pattern, communication with parents, number of brothers and sisters, number of good friends, relationship with teachers and classmates, pocket money, academic pressure and time doing homework. Living arrangements were assessed by asking students which individuals lived in their primary homes. Family economic status was measured by asking students their perceptions of their family’s current economic status (ranging from below average to above average). Parental smoking and drinking pattern was assessed by asking “whether your father/ mother smoke currently”? and “whether your father/mother drink currently”? Family communication was assessed by asking how often students communicated with parents regarding everyday life. The number of brothers and sisters as well as the number of good friends were assessed by a 4-point scale (0, 1, 2, >=3). Relationship with classmates or teachers was assessed by self-ratings from poor to good. Pocket money was assessed by a 3-point scale (<200RMB, 200-499RMB, >500RMB). Academic pressure was assessed by a 3-point scale, from below to above average. Time doing homework was assessed by a 3-point scale (<=1 h, 2−3 h, >3 h).

### Statistical analysis

All data were independently entered by two investigators using EpiData 3.1. Statistical analyses were conducted using SPSS V.21.0, SAS V.9.2 and R V.3.2.2. Differential relationships across genders were assessed separately for boys and girls. Descriptive analyses were used to describe the demographic characteristics and the prevalence of health risk behaviors. Categorical data were reported in the form of frequencies and proportion. Continuous data were reported in the form of means and standard deviations (SDs). Chi-square tests were used to test the difference between the categorical variables. T-test was used to test the difference between the continuous variables. Our study used a multistage sampling method, in which students were grouped into schools. However, the ICC (intra-class correlation coefficient) for boys is 0.009 and for girls is 0.006, indicating a weak group effect. Multiple logistic regression analyses were conducted to investigate the association between smoking, drinking and depressive symptoms. The presence of depressive symptoms was considered the dependent variable, and cigarette smoking and alcohol consumption were the predictor variables. The selection of confounders was based on both priori and empirical considerations. In multiple logistic regression analyses, the following known risk factors were adjusted for: grade, family economic status, parental education level, parental smoking and drinking pattern. The potential confounders were adjusted for based on the change-in-estimate (CIE) criterion with a simulated cutoff yielding a 5% level of type I error (that is, variables that induce a change greater than 95th percentile will be treated as confounders) following Lee^49^. CIE criterion with a simulated cutoff clearly outperformed other criteria in detecting a confounder while yielding the highest accuracy in estimating the true association between exposure and outcome^50^. Two multiplicative interactions were also examined. One included the interaction between cigarette smoking and gender, while the other included the interaction between alcohol consumption and gender. Both interactions were statistically significant. We then performed the multiple logistic regression analysis, stratified by sex. All statistical tests were two-sided, probability values of <0.05 were considered statistically significant. Odds Ratios (ORs) and 95% Confidence Intervals (CIs) were calculated. OR>1 with *P* < 0.05 was reported as a risk factor. Nonresponse to a particular question was treated as missing for all analyses using that variable.

## Additional Information

**How to cite this article**: Yue, Y. *et al.* Gender differences in the association between cigarette smoking, alcohol consumption and depressive symptoms: a cross-sectional study among Chinese adolescents. *Sci. Rep.* **5**, 17959; doi: 10.1038/srep17959 (2015).

## Figures and Tables

**Table 1:** Sample characteristics by depressive symptoms, smoking, drinking and gender (*N* = 18496).

Variables	Total, n (%)	Depressive symptoms, n (%)	Cigarette smoking, n (%)	Alcohol consumption, n (%)	CIE^1^,%	CIE^2^, %	Girls, n (%)	Boys, n (%)	P value*
Total	18496(100)	1685(9.1)	1636(8.8)	4340(23.5)			9731(52.6)	8765(47.4)	
Age(year)**	15.5 ± 1.7	15.6 ± 1.6	16.3 ± 1.4	15.8 ± 1.6			15.5 ± 1.7	15.4 ± 1.7	<0.001
Cigarette smoking	1636(8.8)	**218(13.3)**		**1101(67.3)**		−3.9	186(11.4)	1450(88.6)	<0.001
Alcohol consumption	4340(23.5)	**599(13.8)**	**1101(25.4)**		−25.9		1580(36.4)	2760(63.6)	<0.001
Depressive symptoms	1685(9.1)		**218(12.9)**	**599(35.5)**			1012(60.1)	673(39.9)	<0.001
Gender					25.0	10.9			
Girls	9731(52.6)	**1012(10.4)**	**186(1.9)**	**1580(16.2)**					
Boys	8765(47.4)	**673(7.7)**	**1450(16.5)**	**2760(31.5)**					
Grade					−4.1	−2.7			<0.001
7th	3213(17.4)	**212(6.6)**	**81(2.5)**	**443(13.8)**			1503(46.8)	1710(53.2)	
8th	4064(22.0)	**375(9.2)**	**223(5.5)**	**846(20.8)**			2052(50.5)	2012(49.5)	
9th	484(2.6)	**59(12.2)**	**34(7.0)**	**112(23.1)**			229(47.3)	255(52.7)	
10th	5788(31.3)	**553(9.6)**	**711(12.3)**	**1558(26.9)**			3164(54.7)	2624(45.3)	
11th	4683(25.3)	**473(10.1)**	**551(11.8)**	**1312(28.0)**			2644(56.5)	2039(43.5)	
12th	264(1.4)	**13(4.9)**	**36(13.6)**	**69(26.1)**			139(52.7)	125(47.3)	
Father’s education level					−1.1	−0.5			<0.001
Primary school or lower	3682(19.9)	**370(10.0)**	329(8.9)	**849(23.1)**			2094(56.9)	1588(43.1)	
High school	13412(72.5)	**1149(8.6)**	1195(8.9)	**3120(23.3)**			6953(51.8)	6459(48.2)	
University or higher	1249(6.8)	**146(11.7)**	104(8.3)	**341(27.3)**			592(47.4)	657(52.6)	
Missing data	153(0.8)								
Mother’s education level					0.7	0.2			<0.001
Primary school or lower	5722(30.8)	531(9.3)	**457(8.0)**	**1271(22.2)**			3267(57.1)	2455(42.9)	
High school	11830(64.0)	1050(8.9)	**1095(9.3)**	**2813(23.8)**			6031(51.0)	5799(49.0)	
University or higher	805(4.4)	90(11.2)	**78(9.7)**	**231(28.7)**			355(44.1)	450(55.9)	
Missing data	139(0.8)								
Living arrangement					−0.2	0.0			0.605
Two biological parents	10123(54.7)	**831(8.2)**	876(8.7)	2398(23.7)			5293(52.3)	4830(47.7)	
Only father or mother	3036(16.4)	**355(11.7)**	276(9.1)	721(23.7)			1617(53.3)	1419(46.7)	
Others	5268(28.5)	**495(9.4)**	482(9.1)	1214(23.0)			2781(52.8)	2487(47.2)	
Missing data	69(0.4)								
Number of brothers and sisters					1.1	1.1			<0.001
0	6751(36.5)	**602(8.9)**	**653(9.7)**	**1739(25.8)**			3009(44.6)	3742(55.4)	
1	8316(45.0)	**710(8.5)**	**743(8.9)**	**1895(22.8)**			4589(55.2)	3727(44.8)	
2	2395(12.9)	**246(10.3)**	**162(6.8)**	**474(19.8)**			1543(64.4)	852(35.6)	
>=3	991(5.4)	**124(12.5)**	**77(7.8)**	**222(22.4)**			570(57.5)	421(42.5)	
Missing data	43(0.2)								
Family economic status					−0.9	1.1			<0.001
Above average	1944(10.5)	**128(6.6)**	**202(10.4)**	493(25.4)			837(43.1)	1107(56.9)	
Average	10617(57.4)	**853(8.0)**	**883(8.3)**	2482(23.4)			5690(53.6)	4927(46.4)	
Below average	5857(31.7)	**700(12.0)**	**548(9.4)**	1347(23.0)			3154(53.9)	2703(46.1)	
Missing data	78(0.4)								
Communication with parents					−8.7	−6.2			<0.001
More with father	1418(7.7)	**124(8.7)**	**146(10.3)**	**347(24.5)**			680(48.0)	738(52.0)	
More with mother	5121(27.7)	**390(7.6)**	**350(6.8)**	**1091(21.3)**			3188(62.3)	1933(37.7)	
Much with both	4078(22.0)	**161(3.9)**	**270(6.6)**	**791(19.4)**			2116(51.9)	1962(48.1)	
Little with both	7760(42.0)	**1001(12.9)**	**859(11.1)**	**2091(26.9)**			3695(47.6)	4065(52.4)	
Missing data	119(0.6)								
Relationship with teachers					−3.2	2.2			<0.001
Good	10787(58.3)	**654(6.1)**	**925(8.6)**	2556(23.7)			5659(52.5)	5128(47.5)	
Average	7024(38.0)	**816(11.6)**	**632(9.0)**	1615(23.0)			3779(53.8)	3245(46.2)	
Poor	635(3.4)	**214(33.7)**	**75(11.8)**	158(24.9)			269(42.4)	366(57.6)	
Missing data	50(0.3)								
Relationship with classmates					−10.0	−5.6			<0.001
Good	7374(39.9)	**394(5.3)**	**579(7.9)**	**1641(22.3)**			3732(50.6)	3642(49.4)	
Average	9743(52.6)	**974(10.0)**	**840(8.6)**	**2234(22.9)**			5424(55.7)	4319(44.3)	
Poor	1325(7.2)	**313(23.6)**	**211(15.9)**	**453(34.2)**			552(41.7)	773(58.3)	
Missing data	54(0.3)								
Number of good friends					6.1	7.1			<0.001
0	386(2.1)	**133(34.5)**	**39(10.1)**	**84(21.8)**			169(43.8)	217(56.2)	
1	663(3.6)	**140(21.1)**	**42(6.3)**	**123(18.6)**			391(59.0)	272(41.0)	
2	1898(10.3)	**248(13.1)**	**106(5.6)**	**368(19.4)**			1228(64.7)	670(35.3)	
>=3	15499(83.8)	**1155(7.5)**	**1440(9.3)**	**3749(24.2)**			7918(51.1)	7581(48.9)	
Missing data	50(0.3)								
Pocket money (RMB)					−2.0	−0.5			0.042
<200	13955(75.4)	**1251(9.0)**	**1079(7.7)**	**3006(21.5)**			7297(52.3)	6658(47.7)	
200-499	3684(19.9)	**329(8.9)**	**405(11.0)**	**1049(28.5)**			1998(54.2)	1686(45.8)	
>500	721(4.0)	**91(12.6)**	**142(19.7)**	**261(36.2)**			361(50.1)	360(49.9)	
Missing data	136(0.7)								
Time doing homework					4.6	1.6			<0.001
<=1	6987(37.8)	**530(7.6)**	**917(13.1)**	**1920(27.5)**			3826(54.8)	3161(45.2)	
2-3	8912(48.1)	**769(8.6)**	**507(5.7)**	**1799(20.2)**			3807(42.7)	5105(57.3)	
>3	2507(13.6)	**384(15.3)**	**208(8.3)**	**603(24.1)**			1096(43.7)	1411(56.3)	
Missing data	90(0.5)								
Academic pressure					2.5	−1.0			<0.001
Above average	7859(42.5)	**1119(14.2)**	**671(8.5)**	**1914(24.4)**			4349(55.3)	3510(44.7)	
Average	8109(43.8)	**409(5.0)**	**612(7.5)**	**1754(21.6)**			4381(54.0)	3728(46.0)	
Below average	2489(13.5)	**154(6.2)**	**350(14.1)**	**669(26.9)**			983(39.5)	1506(60.5)	
Missing data	39(0.2)								
Father current smoking	10711(57.9)	973(9.1)	**1009(9.4)**	**2652(24.8)**	−0.7	0.3	5729(53.5)	4982(46.5)	0.006
Mother current smoking	243(1.3)	**35(14.4)**	**35(14.4)**	**77(31.7)**	−1.2	−0.1	131(53.9)	112(46.1)	0.687
Father current drinking	5392(29.2)	**572(10.6)**	**532(9.9)**	**1501(27.8)**	−1.3	−1.8	2740(50.8)	2652(49.2)	0.001
Mother current drinking	158(0.9)	19(12.0)	16(10.1)	**50(31.6)**	−0.5	−0.5	80(50.6)	78(49.4)	0.610

Abbreviations: CIE, change in estimate.

CIE^1^: change in estimate of the association between cigarette smoking and depressive symptoms resulting from including the covariate into the crude model.

CIE^2^: change in estimate of the association between alcohol consumption and depressive symptoms resulting from including the covariate into the crude model.

^*^Chi-square tests were used to test the difference between girls and boys by the above-mentioned categorical variables, and a t-test was used to test the age difference between girls and boys.

^**^Age data presented as the means ± SD, and SD = Standard deviation.

**Bold type** indicates that the correlation is significant (*P* < 0.05) according to Chi-square tests between the above-mentioned categorical variables and smoking, drinking and depression status.

**Table 2:** Odds ratios, adjusted odds ratio and 95% confidence intervals of depressive symptoms among adolescents.

Variables	Total	Girls				Boys		
	Depressive symptoms, n	Depressive symptoms, n	OR (95% CI)	AOR (95% CI)		Depressive symptoms, n	OR (95% CI)	AOR (95% CI)
Cigarette smoking								
No	1467	954	1.0	1.0		513	1.0	1.0
Yes	218	58	**4.1(3.0-5.6)**	**2.3** (**1.6-3.4)**		160	**1.6(1.4-2.0)**	1.2(0.9-1.5)
*P* for interaction: Cigarette smoking × Gender					<0.001			
Alcohol consumption								
No	1086	717	1.0	1.0		369	1.0	1.0
Yes	599	295	**2.4(2.1-2.8)**	**2.1** (**1.8-2.5)**		304	**1.9(1.6-2.2)**	**1.7(1.4-2.1)**
*P* for interaction: Alcohol consumption × Gender					0.007			

Abbreviations: OR, odds ratio by univariate logistic regression. 95% CI, 95% confidence interval. AOR, adjusted odds ratio by multiple logistic regression adjusted for grade, family economic status, parental education level, parental smoking and drinking pattern, communication with parents, number of brothers and sisters, number of good friends, relationship with teachers and classmates, pocket money, academic pressure and time doing homework.

**Bold type** indicates that the CI does not include the null according to logistic regression analyses.

## References

[b1] World Health Organization, WHO calls for stronger focus on adolescent health. WHO Media centre. (2014) Available at: http://www.who.int/mediacentre/news/releases/2014/focus-adolescent-health/en/. (Accessed: 13th September 2015).

[b2] Jane C. E., Erkanli A. & Angold A. Is there an epidemic of child or adolescent depression? J Child Psychol Psychiatry 47, 1263–1271 (2006). 17176381 10.1111/j.1469-7610.2006.01682.x

[b3] Kessler R. C. *et al.* Lifetime Prevalence and Age-of-Onset Distributions of DSM-IV Disorders in the National Comorbidity Survey Replication. Archives of General Psychiatry 62, 593 (2005). 15939837 10.1001/archpsyc.62.6.593

[b4] Verdurmen J., Monshouwer K., van Dorsselaer S., ter Bogt T. & Vollebergh W. Alcohol use and mental health in adolescents: interactions with age and gender-findings from the Dutch 2001 Health Behaviour in School-Aged Children survey. J Stud Alcohol 66, 605–609 (2005). 16329458 10.15288/jsa.2005.66.605

[b5] Khuder S. A., Dayal H. H. & Mutgi A. B. Age at smoking onset and its effect on smoking cessation. Addict Behav 24, 673–677 (1999). 10574304 10.1016/s0306-4603(98)00113-0

[b6] Kann L. *et al.* Youth Risk Behavior Surveillance - United States, 2013. MMWR Surveill Summ 63, 1–168 (2014). 12102329

[b7] Azagba S., Minaker L. M., Sharaf M. F., Hammond D. & Manske S. Smoking intensity and intent to continue smoking among menthol and non-menthol adolescent smokers in Canada. Cancer Cause Control 25, 1093–1099 (2014). 10.1007/s10552-014-0410-6 24913782

[b8] Galaif E. R., Sussman S., Newcomb M. D. & Locke T. F. Suicidality, depression, and alcohol use among adolescents: a review of empirical findings. Int J Adolesc Med Health 19, 27–35 (2007). 17458321 10.1515/ijamh.2007.19.1.27 PMC3134404

[b9] Miller J. W., Naimi T. S., Brewer R. D. & Jones S. E. Binge Drinking and Associated Health Risk Behaviors Among High School Students. Pediatrics 119, 76–85 (2007). 17200273 10.1542/peds.2006-1517

[b10] Mushquash A. R. *et al.* Depressive symptoms are a vulnerability factor for heavy episodic drinking: A short-term, four-wave longitudinal study of undergraduate women. Addict Behav 38, 2180–2186 (2013). 23454875 10.1016/j.addbeh.2012.11.008

[b11] Mendelsohn C. Smoking and depression. Public Health Rep 125, 763 (2010).

[b12] Crum R. M., Storr C. L., Ialongo N. & Anthony J. C. Is depressed mood in childhood associated with an increased risk for initiation of alcohol use during early adolescence? Addict Behav 33, 24–40 (2008). 17587505 10.1016/j.addbeh.2007.05.008 PMC2492760

[b13] Wu P. *et al.* Childhood Depressive Symptoms and Early Onset of Alcohol Use. Pediatrics 118, 1907–1915 (2006). 17079561 10.1542/peds.2006-1221 PMC3072781

[b14] Hermann D., Heinz A. & Mann K. Dysregulation of the hypothalamic-pituitary-thyroid axis in alcoholism. Addiction 97, 1369–1381 (2002). 12410778 10.1046/j.1360-0443.2002.00200.x

[b15] Paperwalla K. N., Levin T. T., Weiner J. & Saravay S. M. Smoking and depression. Med Clin N Am 88, 1483–1494 (2004). 15464109 10.1016/j.mcna.2004.06.007

[b16] Goodman E. & Capitman J. Depressive symptoms and cigarette smoking among teens. Pediatrics 106, 748–755 (2000). 11015518 10.1542/peds.106.4.748

[b17] Munafo M. R. & Araya R. Cigarette smoking and depression: a question of causation. The British Journal of Psychiatry 196, 425–426 (2010). 20513848 10.1192/bjp.bp.109.074880

[b18] Roy K., Parker G., Mitchell P. & Wilhelm K. Depression and smoking: examining correlates in a subset of depressed patients. Aust N Z J Psychiatry 35, 329–335 (2001). 11437806 10.1046/j.1440-1614.2001.00889.x

[b19] Rohde P., Kahler C., Lewinsohn P. & Brown R. Psychiatric disorders, familial factors, and cigarette smoking: II. Associations with progression to daily smoking. Nicotine Tob Res 6, 119–132 (2004). 14982696 10.1080/14622200310001656948

[b20] Dudas R. B., Hans K. & Barabas K. Anxiety, depression and smoking in schoolchildren–implications for smoking prevention. J R Soc Promot Health 125, 87–92 (2005). 15819184 10.1177/146642400512500213

[b21] Levola J., Holopainen A. & Aalto M. Depression and heavy drinking occasions: A cross-sectional general population study. Addict Behav 36, 375–380 (2011). 21216107 10.1016/j.addbeh.2010.12.015

[b22] Acierno R. *et al.* Assault, PTSD, family substance use, and depression as risk factors for cigarette use in youth: Findings from the national survey of adolescents. Journal of Traumatic Stress 13, 381–396 (2000). 10948480 10.1023/A:1007772905696

[b23] McCarty C. A. *et al.* Longitudinal associations among depression, obesity and alcohol use disorders in young adulthood. Gen Hosp Psychiat 31, 442–450 (2009). 10.1016/j.genhosppsych.2009.05.013 PMC2732580 19703638

[b24] Wang J. & Patten S. B. Alcohol consumption and major depression: findings from a follow-up study. Can J Psychiatry 46, 632–638 (2001). 11582825 10.1177/070674370104600708

[b25] Kuo P., Gardner C. O., Kendler K. S. & Prescott C. A. The temporal relationship of the onsets of alcohol dependence and major depression: using a genetically informative study design. Psychol Med 36, 1153 (2006). 16734951 10.1017/S0033291706007860

[b26] Ng M. *et al.* Smoking prevalence and cigarette consumption in 187 countries, 1980-2012. JAMA 311, 183–192 (2014). 24399557 10.1001/jama.2013.284692

[b27] Matheson F. I., White H. L., Moineddin R., Dunn J. R. & Glazier R. H. Drinking in context: the influence of gender and neighbourhood deprivation on alcohol consumption. J Epidemiol Community Health 66, e4 (2012). 21330461 10.1136/jech.2010.112441

[b28] Choi W. S., Patten C. A., Gillin J. C., Kaplan R. M. & Pierce J. P. Cigarette smoking predicts development of depressive symptoms among U.S. adolescents. Ann Behav Med 19, 42–50 (1997). 9603677 10.1007/BF02883426

[b29] Theunissen M., Jansen M. & van Gestel A. Are mental health and binge drinking associated in Dutch adolescents? Cross-sectional public health study. BMC Research Notes 4, 100 (2011). 21463499 10.1186/1756-0500-4-100 PMC3078867

[b30] Lo C. C., Monge A. N., Howell R. J. & Cheng T. C. The Role of Mental Illness in Alcohol Abuse and Prescription Drug Misuse: Gender-Specific Analysis of College Students. Journal of Psychoactive Drugs 45, 39–47 (2013). 23662330 10.1080/02791072.2013.763561

[b31] Frezza M. *et al.* High Blood Alcohol Levels in Women. New Engl J Med 322, 95–99 (1990). 2248624 10.1056/NEJM199001113220205

[b32] Blow F. C. & Barry K. L. Use and misuse of alcohol among older women. Alcohol Res Health 26, 308–315 (2002). 12875042 PMC6676682

[b33] Prescott E., Hippe M., Schnohr P., Hein H. O. & Vestbo J. Smoking and risk of myocardial infarction in women and men: longitudinal population study. BMJ 316, 1043–1047 (1998). 9552903 10.1136/bmj.316.7137.1043 PMC28505

[b34] Guo L. *et al.* Prevalence and correlates of sleep disturbance and depressive symptoms among Chinese adolescents: a cross-sectional survey study. Bmj Open 4, e5517 (2014). 10.1136/bmjopen-2014-005517 PMC4120320 25079937

[b35] Wang H. *et al.* The nonmedical use of prescription medicines among high school students: a cross-sectional study in Southern China. Drug Alcohol Depend 141, 9–15 (2014). 24875678 10.1016/j.drugalcdep.2014.04.004

[b36] Hwang H. & Park S. Sensation seeking and smoking behaviors among adolescents in the Republic of Korea. Addict Behav 45, 239–244 (2015). 25727394 10.1016/j.addbeh.2015.01.041

[b37] Zhang X., Li Y., Zhang Q., Lu F. & Wang Y. Smoking and its risk factors in Chinese elementary and middle school students: A nationally representative sample study. Addict Behav 39, 837–841 (2014). 24583272 10.1016/j.addbeh.2014.01.025

[b38] Choi N. G. & DiNitto D. M. Drinking, smoking, and psychological distress in middle and late Life. Aging Ment Health 15, 720–731 (2011). 21562984 10.1080/13607863.2010.551343

[b39] Dumas T. M., Graham K., Bernards S. & Wells S. Drinking to reach the top: Young adults’ drinking patterns as a predictor of status within natural drinking groups. Addict Behav 39, 1510–1515 (2014). 24968362 10.1016/j.addbeh.2014.05.019

[b40] Myers J. K. & Weissman M. M. Use of a self-report symptom scale to detect depression in a community sample. Am J Psychiatry 137, 1081–1084 (1980). 7425160 10.1176/ajp.137.9.1081

[b41] Radloff L. S., E. The CES-D scale: A self-report depression scale for research in the general populations. Applied Psychological 1, 385–401 (1977).

[b42] Radloff L. S. The use of the Center for Epidemiologic Studies Depression Scale in adolescents and young adults. Journal of Youth and Adolescence 20, 149–166 (1991). 24265004 10.1007/BF01537606

[b43] Hales D. P. *et al.* Factorial validity and invariance of the center for epidemiologic studies depression (CES-D) scale in a sample of black and white adolescent girls. Ethn Dis 16, 1–8 (2006). 16599341

[b44] Camacho P. A. *et al.* [Validity and reliability of the Center for Epidemiologic Studies-Depression scale in Colombian adolescent students]. Biomedica 29, 260–269 (2009). 20128351

[b45] Cheng C. P., Yen C. F., Ko C. H. & Yen J. Y. Factor structure of the Center for Epidemiologic Studies Depression Scale in Taiwanese adolescents. Compr Psychiatry 53, 299–307 (2012). 21621755 10.1016/j.comppsych.2011.04.056

[b46] Lee S. W. *et al.* Factor structure of the Center for Epidemiological Studies Depression Scale in Hong Kong adolescents. J Pers Assess 90, 175–184 (2008). 18444112 10.1080/00223890701845385

[b47] Zhang Jie W. Z. F. G. Development of the Chinese age norms of CES-D in urban area. Chin Ment Health J (In Chinese) 24, 139–143 (2010).

[b48] Yen S., Robins C. J. & Lin N. A cross-cultural comparison of depressive symptom manifestation: China and the United States. J Consult Clin Psychol 68, 993–999 (2000). 11142551 10.1037//0022-006x.68.6.993

[b49] Lee P. H. Is a cutoff of 10% appropriate for the Change-in-Estimate criterion of confounder identification? J Epidemiol 24, 161–167 (2014). 24317343 10.2188/jea.JE20130062 PMC3983286

[b50] Lee P. H. Should we adjust for a confounder if empirical and theoretical criteria yield contradictory results? A simulation study. Sci Rep-Uk 4, 10.1038/srep06085 (2014). PMC5381407 25124526

